# A new graphical format to communicate treatment effects to patients—A web‐based randomized controlled trial

**DOI:** 10.1111/hex.12522

**Published:** 2016-12-16

**Authors:** Jürgen Kasper, Adrian van de Roemer, Jana Pöttgen, Anne Rahn, Imke Backhus, Yasemin Bay, Sascha Köpke, Christoph Heesen

**Affiliations:** ^1^ Department of Health and Caring Sciences Faculty of Health Sciences The Arctic University of Norway Tromsø Norway; ^2^ Division of Internal Medicine University Hospital of Northern Norway Tromsø Norway; ^3^ Unit of Health Sciences and Education MIN Faculty University of Hamburg Hamburg Germany; ^4^ Institute on Didactics in Medicine Michelstadt Germany; ^5^ Department of Neurology Institute for Neuroimmunology and Multiple Sclerosis University Medical Center Hamburg‐Eppendorf Hamburg Germany; ^6^ Institute of Social Medicine and Epidemiology University of Lübeck Lübeck Germany

**Keywords:** evidence based medicine, medical decision making, multiple sclerosis, patient education, patient preference

## Abstract

**Objective:**

Patients making treatment decisions require understandable evidence‐based information. However, evidence on graphical presentation of benefits and side‐effects of medical treatments is not conclusive. The study evaluated a new space‐saving format, CLARIFIG (clarifying risk figures), aiming to facilitate accuracy of comprehension.

**Methods:**

CLARIFIG displays groups of patients with and without treatment benefits as coloured sectors of a proportional bar graph representing in total 100 patients. Supplementary icons indicate the corresponding group's actual condition. The study used an application showing effects of immunotherapy intended to slow disease progression in multiple sclerosis (MS). In a four‐arm web‐based randomized controlled trial, CLARIFIG was compared to the reference standard, multifigure pictographs (MFP), regarding comprehension (primary outcome) and processing time. Both formats were presented as static and animated versions. People with MS were recruited through the website of the German MS society.

**Results:**

Six hundred and eighty‐two patients were randomized and analysed for the primary end point. There were no differences in comprehension rates (MFP_static_=46%, CLARIFIG_static_=44%; *P*=.59; MFP_animated_=23%, CLARIFIG_animated_=30%; *P*=.134). Processing time for CLARIFIG was shorter only in the animated version (MFP_static_=162 seconds, CLARIFIG_static_=155 seconds; *P*=.653; MFP_animated_=286 seconds, CLARIFIG_animated_=189 seconds; *P*≤.001). However, both animated versions caused more wrong answers and longer processing time than static presentation (MFP_static_ vs _animated_: *P*≤.001/.001, CLARIFIG_static_ vs _animated_: *P*=.027/.017).

**Conclusion:**

Comprehension of the new format is comparable to MFP. CLARIFIG has the potential to simplify presentation in more complex contexts such as comparison of several treatment options in patient decision aids, but further studies are needed.

## 1 Background

Patient involvement is particularly indicated in medical decisions comprising more than one option usually including the option of watchful waiting.^1^ Medical reasoning might be capable of comparing treatment efficacy with regard to a defined outcome parameter. The patient's opinion is needed to weigh up the values of different outcomes with potential side‐effects. This applies even more for complexly structured decisions and/or for decisions associated with pronounced scientific uncertainty such as in the case of multiple sclerosis treatments.

Multiple sclerosis (MS) is a chronic inflammatory and degenerative disease starting predominantly in young adults. Apart from symptomatic therapies, the range of treatments comprises an increasing variety of immunotherapeutic options. Making decisions amongst them is challenging with regard to putative risks and uncertain benefit.^2^, ^3^ Comparison of drugs is a complex endeavour as few comparative studies exist and even less evaluating treatment escalation series or long‐term effects of immunotherapies.

**Figure 1:**
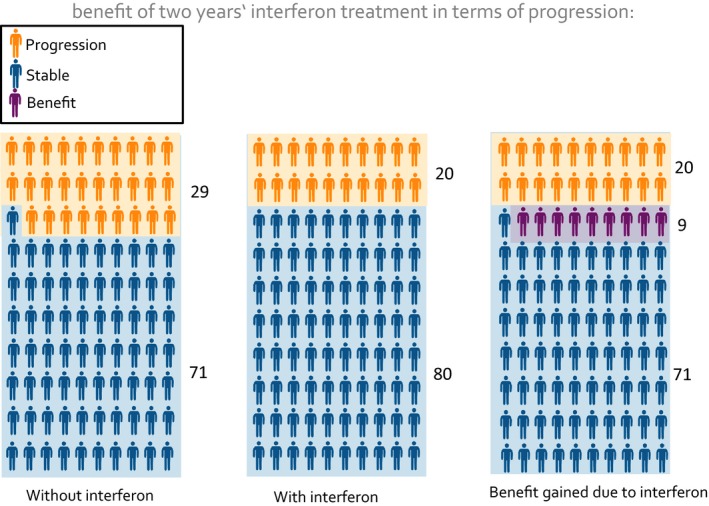
Multiple‐Figure Pictogram: study example [Colour figure can be viewed at wileyonlinelibrary.com]

To be able to make informed choices about immunotherapies, MS patients need information prepared in line with the criteria of evidence‐based patient information.^4^, ^5^ These criteria require communication of benefits and harm for each option presented as changes of absolute risk together with an estimation of the information's trustworthiness. Furthermore, the criteria include presenting event rates by the additional use of graphical frequency formats. Previous studies have shown that different graphical formats visualizing probabilistic information using bar graphs, survival curves and pie charts^4^, ^6^ improve patients’ understanding^7^ and even the quality of physician patient communication^8^, ^9^ when compared to text‐only risk information. Frequently, multiple‐figure pictographs (MFP) (also called icon arrays) are used in evidence‐based patient information as, for example, in decision aids (DA).^4^, ^10^ MFPs show proportions of patients with effects and no effects of a medical intervention using a given reference number of stick figures or smileys (N=100 or N=1000) (Figure 1). MFPs have been proven effective in establishing sustainable comprehension of the difference between relative and absolute risk reduction in MS patients.^11^ Compared to bar graphs, MFPs lead to equal comprehension of the proportions shown. Qualitative evidence suggests that MFPs are better suited to conveying the message of uncertainty about whether or not an individual will belong to the benefit group.^12^ There are, however, practical drawbacks associated with using MFPs, particularly in multiple‐option decisions like those addressed in our previous studies.^13^, ^14^, ^15^ As the number of three consecutive MFPs needed to present the benefit of a single option (Figure 1) multiplies with the number of outcomes reported for benefit and harm and the number of available options, information materials easily become long and difficult to comprehend.^16^ Also, elements of MFPs, that is stick figures or smileys, do not indicate the nature of clinical outcomes (eg in the MS example “disease progression” or “relapses”) and therefore need additional explanations in the graphic's legend. Based on the elaborate qualitative design methodology,^17^ we recently introduced CLARIFIG (clarifying risk figures) combining advantages of both proportional bar graphs and stick figure icons in a new space‐saving format.

This article reports on an investigation aiming to evaluate the new presentation format's efficacy with regard to communicating study effects comprehensibly. Comprehension was defined in terms of accuracy of understanding the given quantities and time needed to process and complete the task. The first research question was: Does CLARIFIG lead to better comprehension and faster processing compared to MFP as the gold standard? Considering the increasing importance of making patient information tools feasible for web‐based presentation, we also aimed at elucidating possible advantages of a stepwise animation. Our second research question was: Does animated presentation lead to better comprehension and faster processing than static presentation?

## 2 Methods

### 2.1 Design

**Figure 2:**
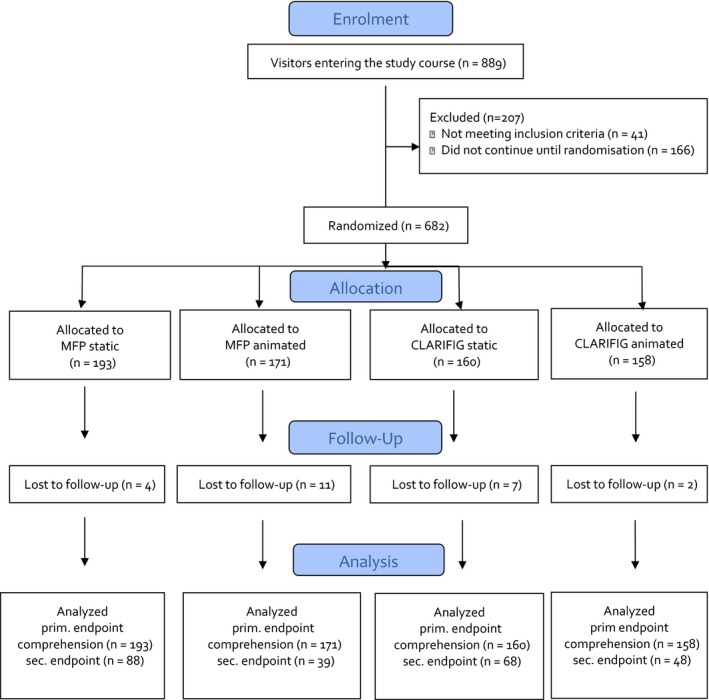
Flowchart [Colour figure can be viewed at wileyonlinelibrary.com]

**Figure 3:**
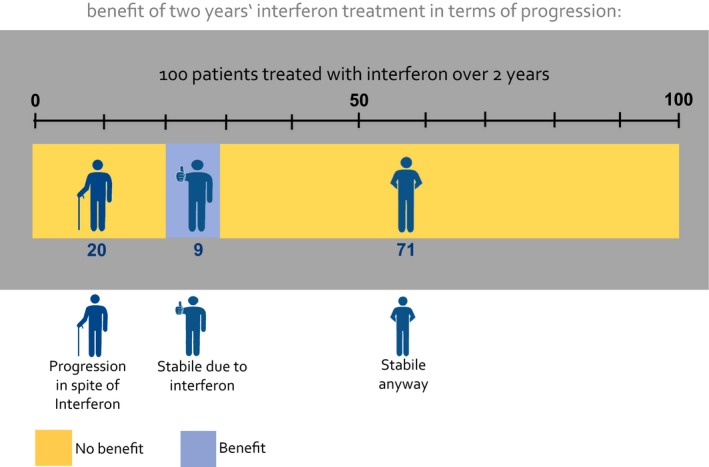
New CLARIFIG graph: study example [Colour figure can be viewed at wileyonlinelibrary.com]

The study used a web‐based four arm randomized controlled trial (Figure 2) using a basic information example considering the effect of interferon‐beta treatment in slowing disease progression in MS.^18^ The previously tested basic example of CLARIFIG (Figure 3) was compared with a corresponding application of the MFP reference standard (Figure 1) and with animated versions of the two graphs, respectively.

The study was part of a research project within the German Multiple Sclerosis Competence network on decision coaching on immunotherapies in MS, which was approved by the Ethics Committee of the Hamburg Chamber of Physicians (PV4576).

### 2.2 Intervention

CLARIFIG presents a sequence of three didactic steps condensed into one proportional bar graph with additional stick figure icons indicating the particular condition of the group represented by each segment of the bar graph (Figure 3). To explain possible results of a treatment option, the following three relevant groups are shown: (i) patients experiencing benefit, (ii) patients who worsen in spite of treatment and (iii) patients who do not benefit because the intended result would have occurred naturally. Applied to the study information example, CLARIFIG shows dichotomous outcome (benefit vs no benefit) indicated by the colour of the bar graph segment and three different types of results as described above: (i) patients remaining stable as a result of immunotherapy treatment, (ii) patients with progression in spite of treatment and (iii) patients who would have remained stable anyway. The patients’ actual clinical condition is additionally indicated by three icons, one with the hands behind the back (indicating stability), one with a thumb up (indicating stability due to treatment) and one with a walking stick (indicating disease progression).

The information displayed in Figure 3 can be summarized by saying that nine of 100 patients benefit (blue bar segment/thumb up) and another 91 do not benefit (yellow segment) but present in two conditions, stable (hands behind the back) and progressed (icon with stick). The study tested the identical application of the CLARIFIG graph previously used during its development.

### 2.3 Sample

To allow for a representative sample of people with MS, we used only two self‐reported inclusion criteria: age ≥18 and a confirmed diagnosis of MS. The sample size was calculated based on the results of the pre‐test. Accordingly, N=143 participants were needed in each group to detect a difference between 10% and 25% of the participants meeting the primary end point. The calculation was based on two‐sided testing with a 5% alpha error and a 90% power. Compensating a 20% dropout rate, this calculation resulted in a proposed sample size of N=686 participants.

### 2.4 Procedure

**Figure 4:**
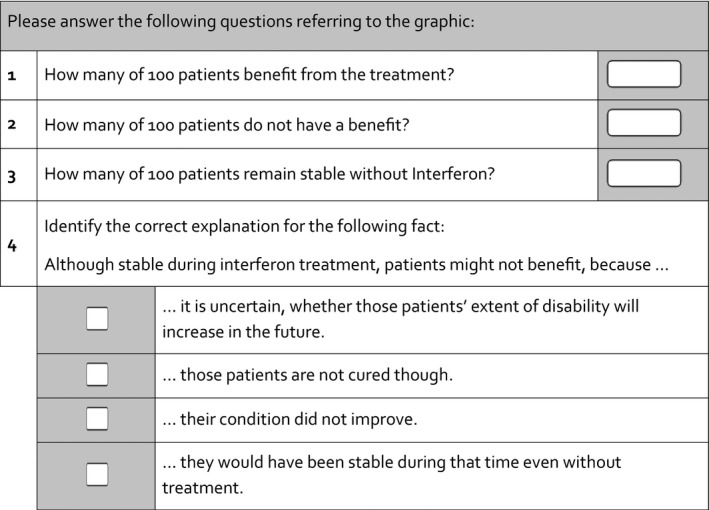
Primary end point

Web presentation of the study was programmed using Unipark software^19^ and accessed from the starting page of the German MS Self‐help Society website (DMSG). It included the following components: invitation teaser, study instructions, the actual intervention consisting of a common introduction and four different presentations of the same information example and common questionnaires. Visitors to the teaser on the DMSG website were invited to participate in a research study about communication of frequencies in patient information materials. Complete anonymity was assured. The explanations about the study aim emphasized usability and comprehensibility of the presentation formats rather than the participants’ performance. Although aware of the existence of various study arms, participants were blinded towards their own allocation. Randomization was conducted individually and documented automatically by a random algorithm within the Unipark software. A second participation via the same IP address was not possible. Participants were free to decide on how much time they wanted to spend on each chart. However, returning to a previous chard was not possible. After entering the study, patients were asked to provide demographic‐ and disease‐related personal data. Briefing the participants for the coming information example, a short presentation (three charts) was then provided. Depending on group allocation, graphical presentations about the benefit of interferon treatment to delay disease progression varied slightly with regard to length (one to three charts) and presentation mode (static vs animated). The primary end point, comprehension, was assessed immediately after display of graphical presentations (Figure 4). To prevent memory effects, display of the respective graph was continued until all questions had been answered. After the completion of the questionnaire, the system registered a participant as a finisher. However, before the procedure was officially finished, participants were additionally asked to fill in a numeracy questionnaire.

### 2.5 Measurements

The primary end point was previously developed and tested as a measure of accurate comprehension of the given quantitative information.^17^ The score was dichotomized, defining four correct answers to the given set of four questions as correct and any other combination as false including missing answers. Beyond the recall of the pure quantity of benefit, the measure requires full comprehension of the complementary frequencies of patients without benefit. Mostly challenging (lowest estimate of item difficulty) was item 4, a multiple‐choice question assessing understanding of the possibility of “no benefit” even though patients remained stable (Figure 4). Our previous qualitative research found the idea that the actual medical result cannot necessarily be equated with benefit to be counterintuitive at first glance and therefore difficult to understand. The secondary end point, processing time, was measured from the start of the study presentation and until completion of the primary end point questionnaire. Systematic variation of the time needed to complete the task was caused only by the presentation format, as all other parts of the study were identical. Differences in processing time were considered important, although the type of hardware used as well as connection speed might have led to individual differences, but no differences between groups were expected due to randomization. Disability was assessed with an eight‐step ordinal measure based on the CAMBS scale.^20^ To assess subjectively perceived cognitive impairment, four ordinally scaled items of the HAQUAMS instrument were applied.^21^ In addition, the questionnaire assessed age, education, disease course, disease duration, medication status and previous participation in related studies. Numeracy was assessed using five of nine dichotomous test items from the Berlin Numeracy scale.^22^

### 2.6 Analyses

Descriptive statistics were used to characterize the sample and the four study groups (Table 1) with regard to demography, disease‐related data and numeracy. In the data matrix used by the statisticians analysing the trial, the nature of the four conditions was disguised. Participants were included in the analyses of the primary end point if they at least reached the place where the four‐item comprehension test was provided. Missing values were counted as “not correct.” Analyses of primary and secondary end points were conducted pairwise within the relevant factor steps. Fisher's exact tests were applied to test for the effects of the frequency format on comprehension separately for the two presentation types. *T* tests for unpaired samples were applied to test for effects of the frequency format on processing time. However, only finishers with correct results were included in this analysis. The impact of the presentation type (static vs animated) was tested separately for the two formats using Fisher's exact tests for comprehension and unpaired *t* tests for processing time.

**Table 1:** Descriptive data from RCT

	MFP static	MFP animated	CLARIFIG static	CLARIFIG animated	Total
n	193	171	160	158	682
Age	39.6 (10.7)	38.0 (10.9)	41.4 (10.9)	41.4 (11.0)	40.1 (10.9)
Disease course					
Early	13 (7%)	15 (9%)	6 (4%)	7 (4%)	41 (6%)
Relapsing remitting	131 (68%)	101 (59%)	103 (64%)	99 (63%)	434 (63.6%)
Secondary chronic	20 (10%)	28 (16%)	25 (16%)	24 (15%)	97 (14.2%)
Primary chronic	14 (7%)	6 (4%)	10 (6%)	9 (6%)	39 (5.7%)
Unclear	15 (8%)	21 (12%)	16 (10%)	19 (12%)	71 (10.4%)
Female	143 (74%)	116 (67.8%)	113 (70.6%)	113 (71.5)	485 (71%)
University‐level education	61 (31.6)	56 (32.7%)	47 (29.4%)	47 (29.4%)	47 (29.7%)
Wheelchair‐dependent	9 (4.7%)	14 (8.2%)	15 (9.4%)	10 (6.3%)	48 (7%)
Cognitive impairment	2.5 (0.9)	2.3 (1.0)	2.3 (1.0)	2.5 (1.0)	2.4 (1.0)
Numeracy	2.14 (1.06)	2.22 (.95)	1.91 (1.1)	1.87 (1.1)	2.04 (1.1)

The influence of numeracy and cognitive impairment was tested using unpaired *t* tests between subgroups of participants meeting and not meeting the primary end point and divided by median split of processing time, respectively.

Moderation of the rate of primary end point achievement by education or disease progression was tested using Fisher's exact tests, moderation of the secondary end point using ANOVA.

All statistical analyses were conducted using IBM Corp. Released 2012. IBM SPSS Statistics for Windows, Version 21.0. Armonk, NY: IBM Corp.

## 3 Results

Of 889 interested visitors, 682 completed the demographic questionnaire, fulfilled the inclusion criteria and were randomized. About 658 completed the study (for demographic data see Table 1) by at least finishing the primary end point task. The rate of dropout was generally low (n=24, 2.7%), but differed slightly between study conditions [MFP_static_ 4 (2.1%), MFP_animated_ 11 (6.4%), CLARIFIG_static_ 7 (4.4%), CLARIFIG_animated_ 2 (1.3%)]. Characteristics of participants were comparable between study groups.

### 3.1 Primary end point

The two formats did not differ with regard to frequencies of comprehension, neither in the static nor in the animated presentation (MFP_static_=46%, CLARIFIG_static_=44%; *P*=.59; animated MFP_animated_=23%, CLARIFIG_animated_=30%; *P*=.134) (Table 2). Single correct answers within the four‐item comprehension questionnaire were more frequent; 85% of the participants identified the correct number of patients benefiting from treatment (Table 2).

**Table 2:** Descriptive results primary end point

Results in the four‐item comprehension test				
Format	MFP		CLARIFIG	
Presentation	Static	Animated	Static	Animated
Sample size	193	171	160	158
Question 1	86%	80%	86%	89%
Question 2	64%	71%	67%	43%
Question 3	86%	39%	91%	90%
Question 4	77%	75%	76%	82%
Total score	88 (46%)	39 (23%)	68 (44%)	48 (30%)

For the static presentation, the animated formats led to significantly less comprehension and longer processing time (MFP: *P*≤.001).

### 3.2 Secondary end point

CLARIFIG showed advantages regarding processing time only in the animated version (MFP_static_=162 seconds. (SD 100), CLARIFIG_static_=156 seconds. (SD 76); *P*=.653; MFP_animated_=286 seconds (SD 172), CLARIFIG_animated_=188 seconds. (SD 62); *P*≤.001). However, compared to the static presentation, the animated formats led to significantly less comprehension and longer processing time (MFP: *p* <= .001 / .001, CALRIFIG: *p* = .027/.017) (Table 3).

**Table 3:** Results for secondary end point: processing time needed

	Processing time			
	MFP	CLARIFIG		*P*
Static presentation				
Time to complete the survey	87162.49(SD: 99.7)	67155.89 (SD: 75.89)	154	.653
Animated presentation				
Time to complete the survey	39285.74 (SD: 172.11)	47188.45 (SD: 62.16)	86	.001

Comprehension was unrelated to processing time in all study groups (static: *P*=.138; animated: *P*=.776). Numeracy was positively related to comprehension (*P*=.016), but had no impact on processing time (static: *P*=.404; animated: *P*=.18). No moderator effects on primary or secondary end points were seen for either cognitive impairment or education level.

## 4 Discussion

This paper describes the testing of a new format for communication of treatment effects to patients composed of a simple proportional bar graph including stick figure icons. Frequency graphs are only one element in a cocktail of essential ingredients of comprehensible patient information. Following the criteria of evidence‐based patient information,^4^ this cocktail also includes, for example, the definitions of possible treatment goals and patient‐relevant outcomes. Other essential elements are a balanced presentation of possible benefits between various medical options and presentation of potential harm alongside presentation of benefits. The complex nature of medical decisions justifies a new format for their presentation. The results of this study clearly show that using the new and condensed format, the quantitative information can be presented as understandably as using the well‐established MFPs.^10^, ^12^, ^23^, ^24^, ^25^, ^26^

However, there was a gap between recognizing and fully understanding the crucial information about the chance of benefiting from treatment. About 85% of participants (irrespective of group affiliation) correctly identified the proportion benefiting (9%), while <50% of participants in all conditions fully understood this figure was clearly below 50% in all conditions. We are not aware of other studies using the latter instead of the former parameter to assess understanding of numerical risk information. However, our choice of the more rigorous parameter as the primary end point reflects our claim to enable patients to make informed choices. As this requires knowledge about both the absolute rate of benefit and the natural course, our end point was meant to assess complete understanding of the graph. This implied, for example, that patients who have not deteriorated do not necessarily belong in the benefit group. We feel that a patient armed with this knowledge would have a good grasp of the options and would even be capable of unmasking a misleading explanation by their physician, for example communicating relative risk reductions only. The knowledge that positive medical results (such as absence of disease progression) can occur naturally, without treatment, is usually not part of standard information. Misleading information on benefit provided by health professionals and the pharmaceutical industry might therefore have contributed to unrealistic expectations regarding treatment effects and to the primary end point's low‐item difficulty (low frequency of correct solutions).^27^ Nevertheless, this rate is still substantially low in the light of a sound development process. Limits in understanding frequency formats could be caused not only by a lack of conclusiveness of the format itself, but also by a lack of fundamental numerical skills in a high percentage of the public.^28^ Besides numeracy, patients’ understanding of graphical risk communication is moderated by other competencies, by pre‐existing knowledge and beliefs.^29^ Participants in our pilot testing reported internal resistance to accepting the information because of the low rate of benefit indicated. Therefore, they tended to interpret the numbers based on their previous beliefs rather than on the figures provided in the graph. This means, in turn, that graphics are only partially capable of compensating for absent skills.^30^

Due to confounding of various moderators potentially impacting on processing time, the secondary end point should be discussed cautiously. Time in this experiment cannot conclusively be attributed to the extent of cognitive burden. As participants were not aware of a time criterion, variance due to individual working styles might have clouded the meaning of the parameter. More rigorous standardization of the end point would on the other hand have been difficult to apply without putting pressure on participants. With regard to the comparison conducted in this study, consideration of processing time as a compound parameter with practical importance seemed to us nevertheless appropriate.

Contrasting the MFP approach, CLARIFIG manages to explain the frequencies without mentioning a placebo condition, which we initially considered essential. However, by following the patients’ reasoning in our qualitative work, we arrived at a much simpler graphical solution than we had assumed would be necessary. A maximum of simplification of the single frequency formats is required to allow for composing clear presentation of comprehensive information. With regard to its concise format, we expect CLARIFIG to improve comprehension accuracy in comprehensive and more complex contexts. As CLARIFIG meets the needs of patients with multiple sclerosis who often have to consider a broad variety of options, we are currently applying the new method to comparative communication of risks and benefits in decisions with up to seven options.^31^ Due to its handy format and intuitive completeness, CLARIFIG is also used for explaining frequencies of benefit and side‐effects in decision aids on the Norwegian platform “Mine Behandlingsvalg.”^32^

The stepwise (“animated”) appearance of the graphic elements used in two of the study conditions obviously confused participants rather than providing meaningful structure. Participants in the animated conditions performed much less well on both comprehension and processing speed than those seeing a stable diagram. Although contradicting our hypotheses, this finding is in line with studies from other authors.^24^, ^33^ Zikmund‐Fischer et al. showed disadvantages of eight animated frequency formats compared with two static presentations. Unanimity of the latter results including ours is important with regard to the increasing availability of web‐based evidence‐based patient information.

The study is strong with regard to large sample size and the low dropout rate, but might be challenged with regard to the representativeness of the study population. Because of the web‐based approach, only patients with a special interest or competence might have accessed the study. Most of the patients in our sample probably were not currently involved in making decisions about immunotherapy, which might have limited the motivation to process the information and might have led to underestimation of the total comprehension rate.

By only looking at two end points (comprehension and processing time), the present study failed to investigate the new graph's possible impact on a number of reasonable end points, such as perception of uncertainty, motivation to take an active role in the decision‐making process, memorability of the information and transfer competence. Most importantly, however, its impact on the decision‐making process in terms of facilitating shared decision making, informed choices and realistic expectation should be focused in further studies.

Effects of frequency formats on risk perception are not yet fully understood,^12^, ^33^ and the optimal format has not yet been found.^6^ Moreover, as the context of the information, the target group and even the numerator size itself moderate the formats’ suitability, current evidence is far from being able to inform systematic recommendations for developers and users of frequency formats.^6^ In this respect, our study responds to a persistent lack of comparative studies and systematic developments in the field of communication and understanding of frequency formats.^6^

In summary, the new format is promising because it has undergone a sound development process involving patients and a rigorous evaluation within a randomized controlled trial. As is immediately evident, CLARIFIG complies with the criteria of evidence‐based patient information,^4^ but also shows practical advantages with regard to multiple‐format arrangements in limited space.

## 5 Conclusion

Comprehension and processing speed of the new format, CLARIFIG, is comparable to commonly used multifigure pictographs (MFPs). The new format is advantageous with regard to space requirements and will facilitate the comparison of different treatment options in comprehensive patient information. This trial is considered exploratory as it compared the methods in a limited application using information from just one isolated study. Having found low comprehension rates irrespective of the experimental condition, the study demonstrates the gap between recognizing and fully understanding the information on the rate of benefit. This result implies that further research is needed on strategies to establish realistic expectations regarding the disease's natural course. Moreover, further studies are needed to prove the format's advantages in more complex contexts such as patient decision aids presenting information on various treatment options in parallel and in other medical domains.

## Authors' Contributions

JK (principal investigator) together with CH, SK and AvdR designed the study and protocol. JK led the development of the graph. The development was conducted together with JP, YB, IB and AvdR, who is responsible for the graphical solutions. JK analysed data, interpreted results and wrote the article with important contributions from CH and SK. All authors contributed to the interpretation of study results and writing of the article.

## Conflict of Interests

JK, AvdR, JP, AR, IB and SK have no conflict of interests. CH has received grants from Biogen, Genzyme Sanofi Aventis, Novartis, Merck‐Serono.
